# Effect of thermally treated barley dietary fiber against hypercholesterolemia

**DOI:** 10.1002/fsn3.1513

**Published:** 2020-04-20

**Authors:** Huma Bader Ul Ain, Farhan Saeed, Muhammad Tauseef Sultan, Muhammad Afzaal, Ali Imran, Vincenzo DeFeo, Calin Cobelschi

**Affiliations:** ^1^ Institute of Home & Food Sciences, Government College University Faisalabad-Pakistan Faisalabad Pakistan; ^2^ Faculty of Rehabilitation and Allied Health Sciences Riphah International University Faisalabad-Pakistan Faisalabad Pakistan; ^3^ Institute of Food Science and Nutrition Bahauddin Zakariya University Multan Pakistan; ^4^ Department of Pharmacy University of Salerno Fisciano Italy; ^5^ Faculty of Medicine Transilvania University of Brasov Brasov Romania

**Keywords:** barley, dietary fiber, functional foods, hypercholesterolemia, thermal modification

## Abstract

Dietary fiber is a nondigestible constituent of vegetal foods, formed by insoluble and soluble dietary fiber. The intake of dietary fiber, especially soluble dietary fiber, is limited and demands researcher's attention. The modification of cereal's dietary fiber, predominantly insoluble fiber, could be one possible solution. The current study evaluated the comparative effects of several thermal treatments on the modification of insoluble dietary fiber in barley and explored their therapeutic potential in vivo against hypercholesterolemia. The two cultivars of barley, Haider‐93 and Jau‐87, were thermally treated using different techniques, and dietary fiber was extracted. Successively, the intake of these dietary fibers was evaluated for its antilipidemic activity in normal and hypercholesterolemic rats. In the first phase, thermal treatments especially cooking without soaking increased the soluble fiber (68.08%). The roasting all increased the soluble fiber contents, however, at relatively lower rate (53.91%). The results of efficacy study revealed that biochemical parameters in control animals were within the normal clinical ranges, thus appraising the safe status of the experimental diets. The thermally treated barley fiber decreased total cholesterol (12.14%–12.63%), low‐density lipoprotein (14.12%–14.85%), and triglycerides (2.25%–4.32%). The study recorded increasing trends for high‐density lipoprotein in both normal and hypercholesterolemic rats. In the nutshell, thermal modification of dietary fiber increased the ratio of soluble to insoluble dietary fiber that improved its hypocholesterolemic potential. The thermally treated barley dietary fiber is effective in reducing the lipid profile in Sprague–dawley rats than untreated dietary fiber and, therefore, can be considered as a functional food and ingredient to cope different lifestyle diseases.

## Graphical abstract

Thermal treatments modify functional properties of barley fibers. Thermally modified barley fibers are active in vivo in hypercholesterolemic rats.

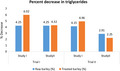

## 1 INTRODUCTION

Functional foods have captured great attention owing to the presence of an array of active ingredients. Among these bioactive compounds, dietary fiber is acknowledged as a major functional ingredient due to its potential to combat different lifestyle disorders (Luithui, Baghya‐Nisha, & Meera, 2019; Zhang, Wang, Cao, & Wang, 2018). Hipsley coined the term “dietary fiber” for first time in 1935, indicating the nondigestible constituents of plant cell walls. Dietary fiber is classified in two classes according to its water solubility, that is, insoluble (IDF) and soluble dietary fiber (SDF). The soluble dietary fiber is effective remedy for reducing the cholesterol, triglyceride, and glucose levels in blood. Therefore, SDF is relatively more functional than IDF. Cereals and other grains contain the higher level of insoluble fiber as compared to soluble fiber. Among cereals, barley contains relatively high soluble amounts of dietary fiber. Even if the cereal fiber is of low cost, its use in foods is considered relatively unsatisfactory due to its poor functionality. Therefore, the need for some modification of its characteristics before incorpo*ratio*n into foods is evident (Borderias, Alonso, & Mateos, 2005; Veronese et al., 2018).

Thermal processes are considered as important approaches for the modification of soluble and insoluble fibers *ratio* and of their physicochemical properties (Zhou, Qian, Zhou, & Zhang, 2012). Different methods are used for thermal modification, such as sterilization, sun drying, steam processing, boiling, frying (mainly deep fat frying), microwave drying, vacuum‐belt drying, roasting, and pressure‐cooking. The steam processing of dietary fibers in *Polygonatum odoratum* revealed the increased oil‐holding capacity, whereas sun drying significantly increased the water‐holding capacity and swelling power (Lan, Chen, Chen, & Tian, 2012). Similarly, continuous vacuum‐belt drying resulted effective in increasing total dietary fiber content in apple pomace at three different temperatures (Yan & Kerr, 2013).

The dietary fiber holds many functional properties that usually correlate with health‐promoting perspectives. The soluble fibers due to their higher water absorption capacities form viscous gel that act as sponge structures, thus reducing the digestion and absorption of nutrients. Second, the slow rate of stomach emptying results reduced transit time in small intestine results in cholesterol reduction. Even, some of the dietary fibers act as prebiotics, thus promoting the growth of intestinal bacteria that in return reduced the synthesis of cholesterol in the body. Barley‐derived β‐glucan can positively reduce the total cholesterol, low‐density lipoprotein (LDL), and triglycerides; however, high‐density lipoprotein (HDL) levels remained unchanged (Talati, Baker, Pabilonia, White, & Coleman, 2009. Furthermore, Behall, Scholfield, and Hallfrisch (2004) reported that barley dietary fiber significantly reduced the lipid levels in moderately hypercholesterolemic men and woman.

Therefore, it can be postulated that partial conversion of insoluble into soluble dietary fiber can improve the efficacy of the fiber‐enriched functional products to cope different lifestyle disorders. The aim of the current study was to evaluate the comparative effect of some thermal treatments on the conversion of insoluble into soluble dietary fiber in two barley cultivars and to evaluate their hypocholesterolemic effects in rodent modeling studies.

## 2 MATERIALS AND METHODS

### 2.1 Plant material

Two barley cultivars, Haider‐93 and Jau‐87, were procured from Ayub Agriculture Research Institute (AARI), Faisalabad. The grains were cleaned to remove any debris or field dirt and sealed in polyethylene bags.

### 2.2 Extraction, determination, and modification of dietary fiber

The fiber extraction and fractionation was conducted as reported by Southgate (1977) with slight modifications. The concent*ratio*ns of acidic solution for the extraction of dietary fiber were adjusted to 1.50% as compared to 1.25% used in the previous studies. The contents of IDF, SDF, and total dietary fiber (TDF) were determined according to the enzymatic gravimetric method 991.43 of AOAC (2005). For modification, grains were ground through a plate mill, obtaining the whole flour (WF). After grinding, WF was sieved, boiled, and cooked using pressure cooker. In the last stage, both barley cultivars were roasted (Pushparaj & Urooj, 2011). All these samples were collected separately to check the influence of each processing stage. Later, the four thermal treatments, that is, soaking, cooking and soaking, cooking of nonsoaked barley, and canning were applied following the protocols by Kutos, Golob, Kac, and Plestenjak (2003).

### 2.3 Extraction of dietary fiber

After thermal treatments, the extraction and fractionation of dietary fibers was carried out as reported by Southgate (1977) with some modifications. The concent*ratio*ns of acidic solution for the extraction of dietary fiber were adjusted to 1.50% as compared to 1.25% used in the previous studies.

### 2.4 Efficacy studies in normal and hypercholesterolemic rats

#### 2.4.1 Animals

Sixty male Sprague–Dawley rats were housed in the Animal Room of Department of Physiology, Government College University, Faisalabad. The research plan was duly approved by “Ethical Departmental Committee” constituted under Office of Research, Innovation, and Commercialization (ORIC) vide Letter No. GCUF/IFHS‐16‐EC‐05. Initially, the rats were acclimatized by feeding basal diet for one week. During the experiment, the environmental conditions were maintained, that is, Tempt: 23 ± 2.0°C, Relative Humidity: 55 ± 5%, 12 hr light–dark period. At the beginning of trial, some rats were dissected to get the baseline values for the selected traits.

### 2.5 Feed plans and housing of experimental rats

In the first phase, three iso‐caloric experimental diets were prepared, that is, control diet contains corn oil (10%), cornstarch (66%), protein (10%), cellulose (10%), mineral (3%), and vitamin mixture (1%). Mineral and vitamin mixture were prepared according to AIN guidelines. The two experimental diets (T_1_ and T_2_) were prepared by replacing 2.0% cellulose with raw barley dietary fiber (2%) and treated barley dietary fiber (2%), respectively. In the second phase, high cholesterol diet, that is, 1.5% of cholesterol along with cholic acid @ 0.5% was given to induce hypercholesterolemia. Periodic examination of rats was carried out to assess the induction of hypercholesterolemia. Rest of the diet plan was same as that of normal rats and diets were provided to the rats concurrently to synchronize their effect on the respective group. At the end of study (42th day), rats were decapitated, after 12 hr of administered fasting, and blood samples were collected in EDTA‐coated tubes. Furthermore, the serum was separated after centrifuging the blood (Rotrofix 32‐A Heltich) for 6 min at 4,042 *g*. The collected sera samples were kept for biochemical evaluation through Rendox Toerauta (RX‐Monza Republic of Ireland).

### 2.6 Serum lipid parameters

The serum lipid profiling with special reference to cholesterol, low‐density lipoproteins (LDL), high‐density lipoproteins (HDL), and triglycerides (TG) was carried out at mentioned intervals. The cholesterol level of collected sera was measured by liquid cholesterol CHOD–PAP method according to Kim et al. (2011). Serum low‐density lipoproteins (LDL) were estimated following the protocol of McNamara, Cohn, Wilson, and Schaefer (1990). Accordingly, the high‐density lipoproteins (HDL) were assessed by cholesterol precipitation method (Alshatwi et al., 2010).The triglycerides were measured by liquid triglycerides (GPO–PAP) method as previously described (Kim et al., 2011).

### 2.7 Statistical analysis

The research trial was repeated twice, and the results were analyzed statistically to draw conclusive inferences. The data obtained for each parameter were subjected to analysis of variance (ANOVA) to determine the level of significance. The means were compared using least significance test (LSD test) to check the variability among diets (Steel et al., 1997).

## 3 RESULTS AND DISCUSSION

### 3.1 Dietary fiber content of barley before and after treatment

The soluble and insoluble fiber contents of the native and the thermally modified barley of the two cultivars were measured and presented as g/100 g dry matter (Table 1). The soluble dietary fiber content was higher in Haider‐93 (5.70 g/100g dm) than in Jau‐87 (4.73 g/100g dm). In comparison, the content of insoluble dietary fiber was higher in Jau‐87 (12.00 g/100g dm) than in Haider‐93 (12.40 g/100g dm). Beloshapka, Buff, Fahey, and Swanson (2016) also reported similar findings that barley contained about 8.6%, 4.8%, and 13.4% insoluble, soluble, and total dietary fiber, respectively.

**Table 1:** Mean values for dietary fiber content of thermally treated barley varieties

Treatments	Jau−87			Haider−93		
	SDF	IDF	TDF	SDF	IDF	TDF
Control	4.73^g^	12.40^a^	17.13^c^	5.70^g^	12.00^a^	17.70^e^
Boiling	7.15^c^	9.37^g^	16.52^d^	8.65^h^	9.07^h^	17.72^e^
Pressure‐cooking	5.36^b^	11.31^b^	18.59^a^	6.12^b^	10.82^b^	16.94^a^
Roasting	7.28^f^	11.31^b^	16.67^d^	8.73^f^	11.00^c^	19.73^g^
Soaking	6.67^d^	9.48^f^	16.15^e^	7.92^c^	9.15^g^	17.07^f^
Cooked–soaked barley	6.39^d^	10.91^c^	17.3^b^	7.61^e^	10.68^d^	18.29^c^
Cooked–nonsoaked barley	7.95^a^	10.48^e^	18.43^a^	9.05^a^	10.07^f^	19.12^b^
Canning	6.39^e^	10.80^d^	17.13^c^	7.69^d^	10.38^e^	18.07^d^

Note

Abbreviations: IDF, Insoluble dietary fiber; SDF, Soluble dietary fiber; TDF, Total dietary fiber.

Means carrying same letters are significantly identical.

The results regarding the modification of barley dietary fiber through thermal treatments have been already published (Bader Ul Ain et al., 2019). The amount of soluble dietary fiber produced is highly dependent on the temperature of the processes (Zhou et al., 2012). The results showed that modification was not significant (*p* = .05) in pressure‐cooking modified. After the1st phase, roasting significantly increased the soluble dietary fiber while boiling caused a significant decrease in the insoluble dietary fiber content in all wet and dry heat treatments. Among all treatments, cooking without soaking out performed rest of the treatments. It was apparent that thermal processes can change the *ratio* of soluble and insoluble fibers (Bader Ul Ain et al., 2019). The high temperature breaks the glycosidic bonds of polysaccharides that can lead to the release of oligosaccharides and thus increase the quantity of soluble dietary fiber (Yi, Wang, Zhuang, Pan, & Huang, 2014). These changes in dietary fibers may have important physiological effects.

#### 3.1.1 Efficacy studies

##### 3.1.1.1 Total cholesterol

**Figure 1:**
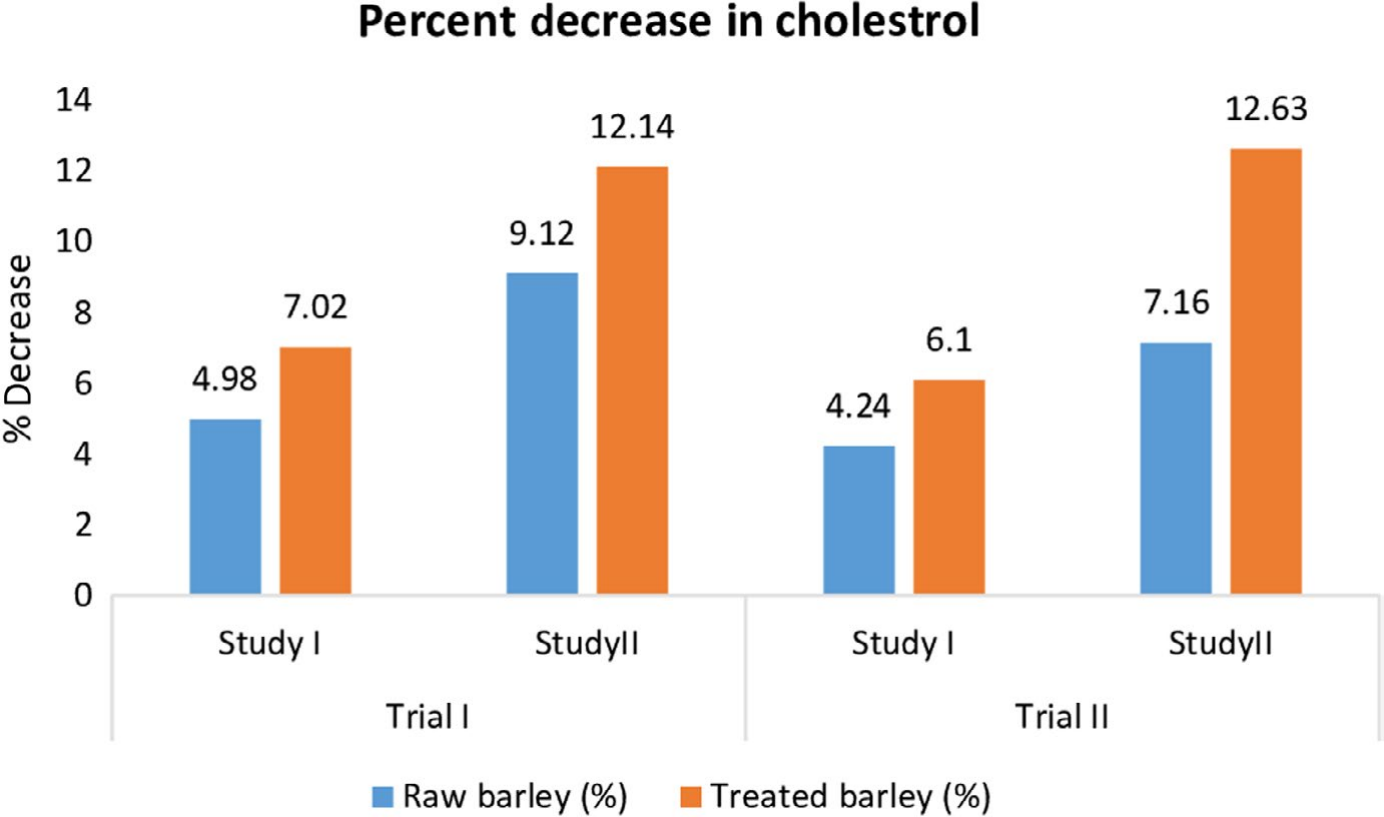
Effect of thermally treated barley fiber on % decrease in cholesterol

The effects of raw and treated barley dietary fiber on lipoprotein profile, such as total cholesterol, LDL, HDL, and triglycerides, were also evaluated. Statistical results revealed that the treatments were varied highly significantly in both trials, while nonsignificant results were shown by variation during the trial I and trial II. Table 2 showed mean values of the effect of raw and treated barley dietary fiber on cholesterol level. In control group (T_0_), maximum cholesterol levels (145.03 ± 7.25 mg/dl and 144.89 ± 7.24 mg/dl) were observed in trial I and trial II of study II (antihypercholesterolemic study). In normal rats, cholesterol level varied from 83.01 ± 4.15 mg/dl to 82.5 ± 4.13 mg/dl. Furthermore, in case of T_1_ (raw barley dietary fiber), a significant decline in cholesterol levels was observed in both studies. In T_1_, cholesterol levels were reduced to 80.23 ± 4.01, 80.98 ± 4.05, 136.86 ± 6.84, and 138.36 ± 6.92 mg/dl in trial I and II of normal study and trial I and II of hypercholesterolemic study, respectively. Maximum decline in cholesterol level was observed in T_2_ (treated barley dietary fiber) as shown in Figure 1. In trial I and II of both studies, cholesterol levels were reduced to 79.45 ± 3.97, 80.22 ± 4.01, 134.35 ± 6.72, and 133.83 ± 6.69 mg/dl, respectively.

**Table 2:** Effect of treatments on cholesterol level

Trials	T_0_	T_1_	T_2_
Study I			
Trial I	82.5 ± 4.13 ^a^	80.23 ± 4.01 ^b^	79.45 ± 3.97 ^c^
Trial II	83.01 ± 4.15 ^a^	80.98 ± 4.05 ^b^	80.22 ± 4.01 ^c^
Study II			
Trial I	62.00 ± 3.1 ^a^	58.19 ± 2.91 ^b^	56.75 ± 2.84 ^c^
Trial II	62.5 ± 3.13 ^a^	58.48 ± 2.92 ^b^	56.86 ± 2.84 ^c^

Note

T_0_ = control diet; T_1_ = raw barley; T_2_ = treated barley.

Means carrying same letters are significantly identical.

The results of the present study were in‐line with the work of some other researchers but with slight variations in percent reductions. A study conducted by AbuMweis, Jew, and Ames (2010) evaluated the lipid‐lowering effect of barley beta‐glucan. The barley beta‐glucan significantly reduced the total cholesterol and LDL levels in dose‐dependent manner. Later, Tiwari and Cummins (2011) performed an in vivo study and observed the inverse association of oat and barley beta‐glucan with cholesterol levels. Ghaffarzadegan, Zhong, Hallenius, and Nyman (2018) administrated barley‐based soluble dietary fiber in hypercholesterolemic rats and elaborated that binding of bile acids is major mechanism behind the cholesterol‐lowering effect of barley soluble dietary fiber (Wang & Ellis, 2014). The cholesterol reduction can be linked with higher binding of bile acids that might result in higher excretion of bile acids through feces (Kim & White, 2012). Ranhotra, Gelroth, Leinen, and Bhatty (1998) reported an inverse relation between barley‐derived soluble fiber intake with blood lipids levels in hamster and barley fibers significantly lowered cholesterol level by 16.4%.

#### 3.1.2 Low‐density lipoprotein (LDL)

**Figure 2:**
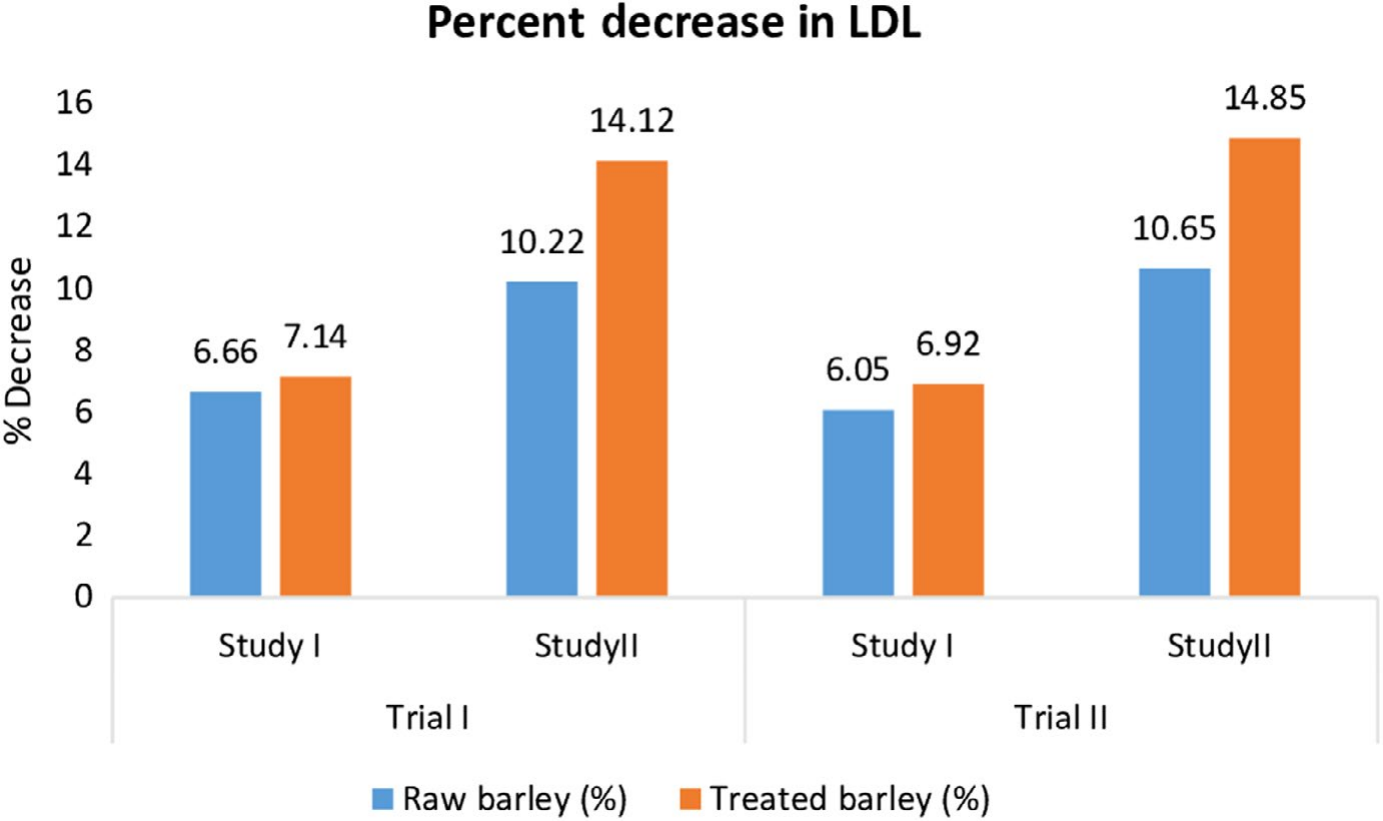
Effect of thermally treated barley fiber on % decrease in LDL

The results revealed that highly significant differences were observed in case of treatments while the impact of trials was nonsignificant (Table 3). Mean values for the effect on low‐density lipoprotein (LDL) level by the raw and treated barley dietary fibers revealed that LDL level was decreased significantly in studies of both trials. The highest LDL level (33.5 ± 1.68 mg/dl) was observed in T_0._ The experimental diets T_1_ (32.42 ± 1.62 mg/dl) and T_2_ (32.33 ± 1.62 mg/dl) decreased the LDL significantly. Likewise, in trial II, the LDL level reduction pattern was same, that is, 33.67 ± 1.68, 32.45 ± 1.62, and 32.22 ± 1.61 mg/dl for T_0_, T_1,_ and T_2_, respectively, in normal study. In antihypercholesterolemic study, the maximum LDL level (62.00 ± 3.1 mg/dl) was observed in control group (animals fed on control or basel diet), followed by LDL level of raw barley dietary fiber fed rats (58.19 ± 2.91 mg/dl), while the least LDL levels were recorded in group of rats fed on treated barley dietary fiber (56.75 ± 2.84 mg/dl) in trial I. Similarly, in trial II, the highest LDL level for normal or control study (62.5 ± 3.13 mg/dl) was exhibited in T_0_ that gradually reduced in T_1_ (58.48 ± 2.92 mg/dl) and then more significantly reduced in T_2_ (56.86 ± 2.84 mg/dl) (Figure 2).

**Table 3:** Effect of treatments on LDL level

Trials	T_0_	T_1_	T_2_
Study I			
Trial I	33.5 ± 1.68 ^a^	32.42 ± 1.62 ^b^	32.33 ± 1.62 ^b^
Trial II	33.67 ± 1.68 ^a^	32.45 ± 1.62 ^b^	32.22 ± 1.61 ^b^
Study II			
Trial I	62.00 ± 3.1 ^a^	58.19 ± 2.91 ^b^	56.75 ± 2.84 ^c^
Trial II	62.5 ± 3.13 ^a^	58.48 ± 2.92 ^b^	56.86 ± 2.84 ^c^

Note

T_0_ = control diet; T_1_ = raw barley; T_2_ = treated barley

Means carrying same letters are significantly identical.

Smith, Queenan, Thomas, Fulcher, and Slavin (2008) evaluated the impact of barley beta‐glucan (0, 3, or 6 g/day) on cholesterol level in human, and it was found that low‐density lipoprotein level was reduced significantly by 10%–13%. The findings of present study are in‐line with the results of Mumford et al. (2011) who demonstrated the effect of dietary fiber consumption on lipoprotein cholesterol levels in premenopausal women with no estradiol intake, and the results revealed that dietary fiber intake produced a decrease in the lipoprotein cholesterol levels. Among lipoprotein cholesterol levels, LDL was reduced significantly. Aman (2006) reported that concentrated barley dietary fiber, that is, beta‐glucan, was found to be significantly effective in reducing LDL levels when the concentrated beta‐glucan‐enriched food products were given to hyperlipidemia patients without metabolic syndrome, and after six weeks of beta‐glucan administ*ratio*n, LDL was lowered by 15%. Talati et al. (2009) worked on the relation of serum lipids and barley‐derived soluble fiber in hypercholesterolemic subjects and found that the use of barley soluble fiber notably reduced the total cholesterol, LDL, and triglycerides, but no effect was probed against HDL.

#### 3.1.3 High‐density lipoprotein (HDL)

**Figure 3:**
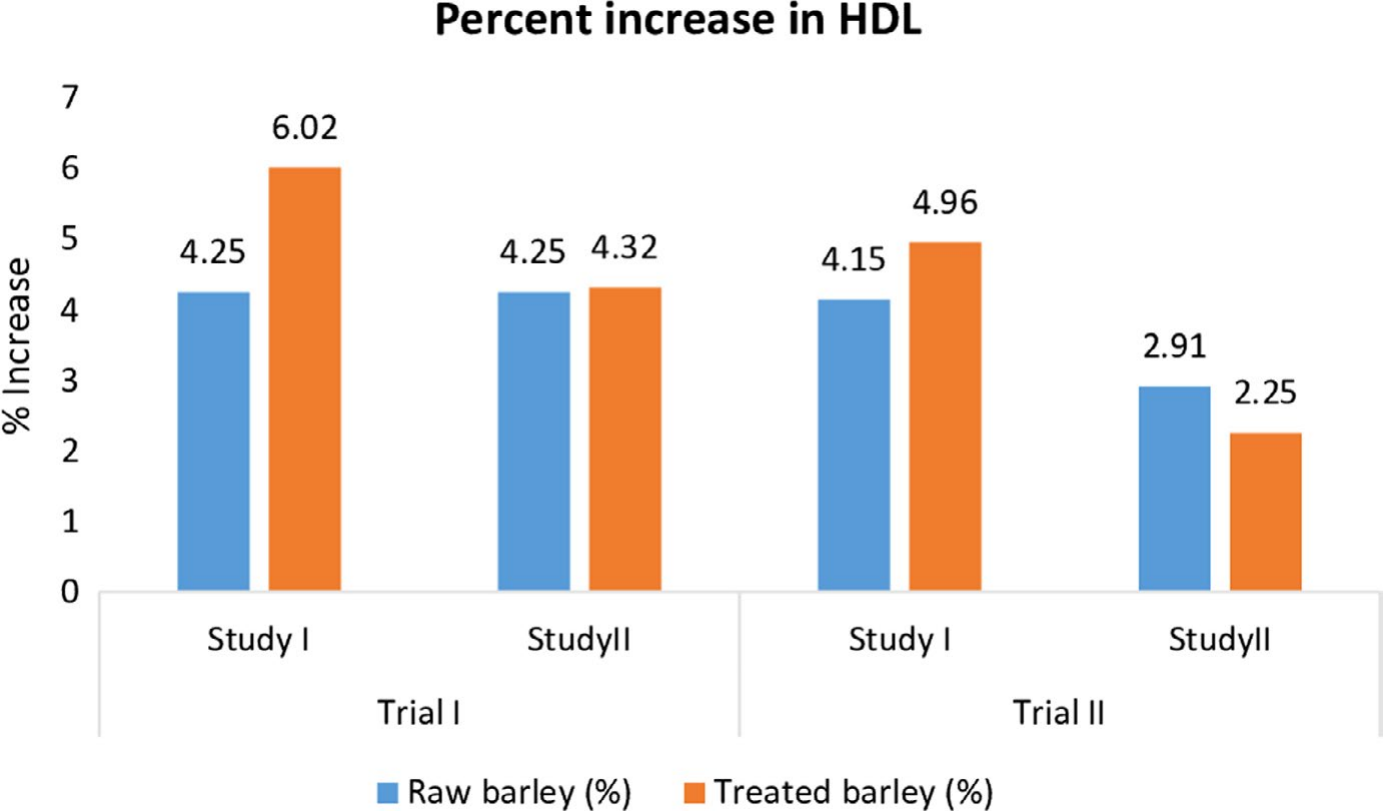
Effect of thermally treated barley fiber on % increase in LDL

The levels of HDL were significantly influenced by treatments (T_0_ = control diet/basal diet, T_1_ = raw barley dietary fiber, and T_2_ = treated barley dietary fiber), but nonmomentous results were shown by trial I and trial II. Mean values and standard deviations for the increasing pattern of HDL level were shown in Table 4. The maximum HDL level (46.24 ± 2.33 mg/dl) was observed in T_2_ of trial II of study II (antihypercholesterolemic study), and the minimum HDL level (36.08 ± 1.80 mg/dl) was observed in T_0_ of trial I of study I. In trial I, the highest HDL level was recorded in T_0_ (36.08 ± 1.80 mg/dl) followed by T_1_ (39.71 ± 1.99 mg/dl) and T_2_ (41.14 ± 2.06 mg/dl) whereas, in trial II, maximum HDL was recorded in T_0_ (36.18 ± 1.81 mg/dl) followed by T_1_ (39.67 ± 1.98 mg/dl) and T_2_ (41.29 ± 2.06 mg/dl) in normal study. Moreover, the maximum HDL (46.05 ± 4.62 mg/dl) was recorded in T2 (treated barley dietary fiber), whereas T0 (control/basal diet) exhibited the lowest value for HDL as 42.13 ± 2.31 mg/dl during trial I. Moreover, values for HDL were documented as 42.03 ± 2.30, 45.06 ± 2.25, and 46.24 ± 2.33 mg/dl for T0, T1, and T3, respectively, in trial II of antihypercholesterolemic study. The highest percent increase was recorded in rats group fed on treated barley dietary fiber in both studies of trial I and trial II (Figure 3).

**Table 4:** Effect of treatments on HDL level

Trials	T_0_	T_1_	T_2_
Study I			
Trial I	36.08 ± 1.80 ^c^	39.71 ± 1.99 ^b^	41.14 ± 2.06 ^a^
Trial II	36.18 ± 1.81 ^c^	39.67 ± 1.98 ^b^	41.29 ± 2.06 ^a^
Study II			
Trial I	42.13 ± 2.31 ^a^	44.47 ± 2.22 ^b^	46.05 ± 4.62 ^a^
Trial II	42.03 ± 2.30 ^b^	45.06 ± 2.25 ^c^	46.24 ± 2.33 ^a^

Note

T0 = control diet; T1 = raw barley; T2 = treated barley.

Means carrying same letters are significantly identical.

Al‐Rewashdeh (2009) checked the effect of barley and wheat on the lipid profile of hypercholesterolemic rats. There was a significant decrease in total cholesterol, LDL, and triglycerides levels, whereas HDL was increased momentously. Wistar rats were fed on barley grain‐derived dietary fiber, and azoxymethane and their effect were monitored on serum lipid levels. The rats fed on barley grain dietary fiber showed a lowered level of LDL, total cholesterol, and triglyceride and increased HDL level, while the azoxymethane fed rats showed significant increase in total cholesterol, LDL, and triglycerides, and HDL level was significantly reduced in this case (Labouar et al., 2011).

#### 3.1.4 Triglycerides

**Figure 4:**
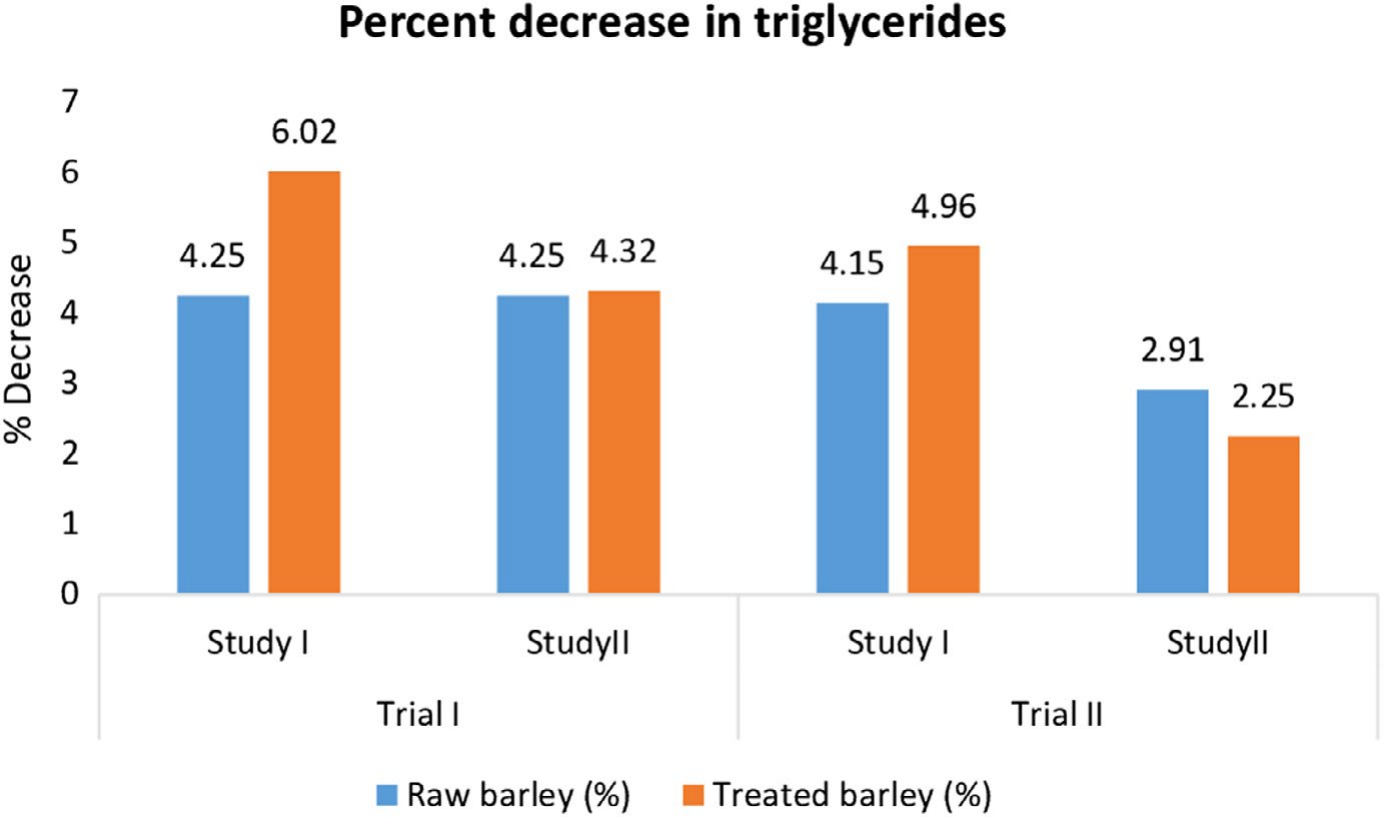
Effect of thermally treated barley fiber on % decrease in triglycerides

Statistical data demonstrated that there was highly significant variation among treatments (Table 5), and triglyceride level showed significant decreasing pattern by the administration of raw and treated barley dietary fibers in rats. In trial I of normal study, findings for triglycerides were observed as 68.51 ± 3.43, 66.9 ± 3.35, and 66.24 ± 3.31 mg/dl for T_0_, T_1,_ and T_2,_ respectively. Similarly, values for triglyceride content of serum were recorded as 68.53 ± 3.43, 67.26 ± 3.36, and 66.49 ± 3.32 mg/dl for T_0_, T_1,_ and T_2,_ respectively, in trial II of normal study. Moreover, in antihypercholesterolemic study, the maximum triglycerides 114.52 ± 5.73 mg/dl were recorded in T_0_ (control diet), whereas T_2_ exhibited lowest value for triglycerides (111.74 ± 5.59 mg/dl) during trial I. Likewise, in trial II, highest triglycerides level was observed in T_2_ (119.94 ± 5.99 mg/dl) followed by triglycerides levels in T_1_ (111.61 ± 5.58 mg/dl) and T_0_ (113.52 ± 5.68 mg/dl) (Figure 4).

**Table 5:** Effect of treatments on triglycerides level

Trials	T_0_	T_1_	T_2_
Study I			
Trial I	68.51 ± 3.43 ^a^	66.9 ± 3.35 ^b^	66.24 ± 3.31 ^c^
Trial II	68.53 ± 3.43 ^a^	67.26 ± 3.36 ^b^	66.49 ± 3.32 ^c^
Study II			
Trial I	114.52 ± 5.73 ^a^	11.74 ± 5.59 ^b^	111.74 ± 5.59 ^b^
Trial II	113.52 ± 5.68 ^a^	111.61 ± 5.58 ^c^	119.94 ± 5.99 ^b^

Note

T0 = control diet; T1 = raw barley; T2 = treated barley.

Means carrying same letters are significantly identical.

Ranhotra et al. (1998) reported that the consumption of barley‐derived soluble fiber significantly reduced the total cholesterol level, triglycerides, and LDL through its binding capacity with bile acids. Through this binding, reabsorption of bile acids in intestine was prevented and these were carried to colon and excreted. The results of present study were in accordance with the results of previous researches (Aman, 2006; Talati et al., 2009).

## 4 CONCLUSION

The studied cultivars of barley were rich in insoluble dietary fiber but are a poor source of soluble dietary fiber. The application of thermal treatments increased the soluble dietary fiber, thus decreasing the concentrations of insoluble dietary fiber. Among treatments, cooking without soaking and roasting were most effective thermal modification with commercial significance. The thermally modified dietary fiber decreased the circulating lipids (total cholesterol, LDL, and triglycerides), thus could be suitable dietary strategy to reduce hypercholesterolemia. The thermally modified barley fiber must be tested for its health‐promoting perspective especially in gastrointestinal tract disorders, diabetes mellitus, and metabolic syndrome. The large‐scale efficacy of modified dietary fiber must be checked in human subjects through clinical trials before warranting their commercial applications.

## CONFLICT OF INTEREST

Authors declare no competing financial interests.

## ETHICAL STATEMENT

The authors declare that they do not have any conflict of interest. The research plan was duly approved by “Ethical Departmental Committee” constituted under Office of Research, Innovation, and Commercialization (ORIC) vide Letter No. GCUF/IFHS‐16‐EC‐05. Written informed consent of the participants was not applicable in the present research study.
